# A biologging database of juvenile white sharks from the northeast Pacific

**DOI:** 10.1038/s41597-022-01235-3

**Published:** 2022-04-01

**Authors:** John O’Sullivan, Christopher G. Lowe, Oscar Sosa-Nishizaki, Salvador J. Jorgensen, James M. Anderson, Thomas J. Farrugia, Emiliano García-Rodríguez, Kady Lyons, Megan K. McKinzie, Erick C. Oñate-González, Kevin Weng, Connor F. White, Chuck Winkler, Kyle S. Van Houtan

**Affiliations:** 1 Monterey Bay Aquarium, Monterey, California 93940 USA; 2 Department of Biological Sciences, California State University, Long Beach, California 90815 USA; 3 Department of Biological Oceanography, CICESE, Ensenada, Mexico; 4 Monterey Bay Aquarium Research Institute, Moss Landing, California 95039 USA; 5 Biological Sciences, Autonomous University of Nuevo León, San Nicolás de los Garza, 66450 Mexico; 6 Hopkins Marine Station, Stanford University, Stanford, California 93950 USA; 7 Aquatic Research Consultants, San Pedro, California USA; 8 Nicholas School of the Environment, Duke University, Durham, North Carolina 27708 USA; 9 Present Address: Institute of Marine Sciences, University of California, Santa Cruz, California 95064 USA; 10 Present Address: Alaska Ocean Observing System, Anchorage, Alaska 99501 USA; 11 Present Address: Georgia Aquarium, Atlanta, GA 30313 USA; 12 Present Address: Virginia Institute of Marine Science, College of William & Mary, Gloucester Point, Virginia USA; 13 Present Address: Department of Organismic and Evolutionary Biology, Harvard University, Cambridge, Massachusetts USA; 14 Present Address: Loggerhead Marinelife Center, Juno Beach, Florida 33408 USA

**Keywords:** Animal migration, Ichthyology, Marine biology

## Abstract

Species occurrence records are vital data streams in marine conservation with a wide range of important applications. From 2001–2020, the Monterey Bay Aquarium led an international research collaboration to understand the life cycle, ecology, and behavior of white sharks (*Carcharodon carcharias*) in the southern California Current. The collaboration was devoted to tagging juveniles with animal-borne sensors, also known as biologging. Here we report the full data records from 59 pop-up archival (PAT) and 20 smart position and temperature transmitting (SPOT) tags that variously recorded pressure, temperature, and light-level data, and computed depth and geolocations for 63 individuals. Whether transmitted or from recovered devices, raw data files from successful deployments (*n* = 70) were auto-ingested from the manufacturer into the United States (US) Animal Telemetry Network’s (ATN) Data Assembly Center (DAC). There they have attributed a full suite of metadata, visualized within their public-facing data portal, compiled for permanent archive under the DataONE Research Workspace member node, and are accessible for download from the ATN data portal.

## Abstract (summary)

**Table:** 

Measurement(s)	animal migration, animal movements, environmental data
Technology Type(s)	biologging, telemetry tags, satellite transmitters
Factor Type(s)	depth • temperature • longitude • latitude
Sample Characteristic - Organism	Carcharodon carcharias
Sample Characteristic - Environment	ocean, nearshore marine environment, coastal aquatic habitat
Sample Characteristic - Location	State of Baja California • State of California • North East Pacific Ocean

## Background & Summary

The biologging and biotelemetry revolution that began with isolated, descriptive case studies^1–5^ has since grown into wide-ranging research disciplines with applications across marine science^6–8^. The Monterey Bay Aquarium (“Aquarium”) has been active in biologging research, helping to pioneer studies across diverse marine taxa, including tunas, sharks, devil rays, sunfish, sea otters, and jellyfish^3,4,9–11^. Over two decades ago, the Aquarium initiated the Juvenile White Shark Project (“Project”), spurred on by the first successful deployment of a satellite transmitting tag on a juvenile white shark (*Carcharodon carcharias*) in 2000^12^. At this time there were several active research programs devoted to understanding adult white sharks, however, none were focused on the juvenile demographic of these apex ocean predators during this important developmental phase. As a result, the Aquarium began an international collaboration devoted to the comprehensive study of juvenile white sharks in the northeastern Pacific.

This collaboration to date has resulted in many scientific publications and graduate student projects^13–22^, each study arising from the analysis of a subset of the overall dataset presented here. The published findings about the biology and ecology of juvenile white sharks initially provided vital insights to the Aquarium’s husbandry team that enabled their successful white shark exhibition (2004–2011). The Aquarium’s exhibitions of juvenile white sharks aimed to inspire ocean conservation through promoting public awareness of the important ecological role played by white sharks as well as the threats sharks face in the wild. Through 2020, the Project deployed 79 electronic tags on 63 juvenile white sharks that have helped document their seasonal migrations and oceanographic preferences, fisheries interactions, nursery locations, ontogenetic shifts, and habitat shifts arising from ocean warming.

Beyond its own programmatic and scientific achievements, through knowledge sharing and funding, the Project helped launch additional juvenile white shark research programs in Australia (Commonwealth Scientific and Industrial Research Organization, principal investigator Barry Bruce), Mexico (Centro de Investigación Científica y de Educación Superior de Ensenada, principal investigator Oscar Sosa-Nishizaki), and southern California (California State University Long Beach, principal investigator Chris Lowe). This collaborative network, within the region and beyond, contributed significantly to the Project’s success and longevity. Beyond the scientific community, the trust and support of countless commercial fisherman was a key element that contributed immeasurably to the Project, particularly in the program’s early development. Throughout, this Project was a privately funded research program, whose present and future status was adversely affected by the economic hardships arising from the COVID-19 pandemic and resulting budgetary reductions.

Here, we provide an extensively curated dataset from electronic tags affixed to juvenile white sharks in the northeast Pacific Ocean. These data were collected from two biotelemetry platforms (details below) that potentially recorded pressure, temperature, and light-level data and additionally computed depth and geolocation information. These data were auto-ingested, post-processed, annotated, and hosted on the United States (US) Animal Telemetry Network (ATN) Data Assembly Center (DAC), where they are publicly accessible. The ATN DAC is an essential part the Integrated Ocean Observing System (IOOS) network of the National Oceanic and Atmospheric Administration (NOAA) National Ocean Service (NOS).

## Methods

### Tagging deployments and study subjects

Table 1 contains an overview of the fields in the metadata file (JWS_metadata.xlsx) providing extensive background details on each of the 79 tag deployments and 63 study subjects. The data in this file give essential contextual information needed to understand the methodological, environmental, and demographic factors surrounding the deployments, which are critical for further examination and hypothesis testing of the sensor data. These metadata fall into several specific categories, but are not limited to, (i) information on the deployed electronic devices (platform, model, Platform Transmitter Terminal identifications), (ii) sharks (unique identifying numbers, sex, length), (iii) capture event (date, location, duration, methodology, interaction type), and (iv) the reporting period (duration, linear surface travel distance).

**Table 1:** Metadata descriptions of the sharks, tagging operations, and deployments for all tags included in the database.

COLUMN HEADER	COLUMN DESCRIPTION
SHARK_ID	unique identification number for each individual tagged shark
DATE_CAP	date of original capture
DAYS_CAP	the number of days between original capture and release
DATE_START	the first date when electronic tags begin recording data
PAT_END	the last date when PAT tag recorded data, when the tag popped and released from the shark: “ND” tag not deployed, “DNT” tag did not transmit
SPOT_END	the last date when SPOT tag recorded data: “ND” tag not deployed, “DNT” tag did not transmit
DEPLOY_DAYS	the maximum deployment duration in days for any applied electronic tag
PAT_MODEL	Wildlife Computers model number for the PAT tag: “ND” tag not deployed
PAT_ID	the unique serial number of the PAT tag: “ND” tag not deployed
PAT_PTT	the PTT number for the PAT tag: “ND” tag not deployed
PAT_DEPLOY_ID	user friendly unique PAT tag deployment ID, researcher assigned, matches ID listed w/in WC data files: “ND” tag not deployed
PAT_MANUFACTURER_ID	manufacturer assigned unique PAT tag deployment ID, ATN internal reference ID: “ND” tag not deployed, “DNT” tag did not transmit
SPOT_MODEL	Wildlife Computers model number for the SPOT tag: “ND” tag not deployed
SPOT_ID	the unique serial number of the SPOT tag: “ND” tag not deployed
SPOT_PTT	the PTT number for the SPOT tag: “ND” tag not deployed
SPOT_DEPLOY_ID	unique SPOT tag deployment ID, matches ID listed w/in WC data files: “ND” tag not deployed
SPOT_MANUFACTURER_ID	manufacturer assigned unique SPOT tag deployment ID, ATN internal reference ID: “ND” tag not deployed, “DNT” tag did not transmit
ACOUSTIC_ID	the identification number of the acoustic tag: “ND” tag not deployed
PAT_RECOVERY	was the PAT tag physically recovered? “YES” it was, “NO” it was not, “ND” tag not deployed
PAT_DATA_TRANS	did the PAT tag transmit it’s data via satellite connection? “YES” it did, “NO” it did not, “ND” tag not deployed
SPOT_DATA_TRANS	did the SPOT tag transmit data via satellite connection? “YES” it did, “NO” it did not, “ND” tag not deployed
DATA_BINNED	for PAT tags, were the tags programmed to transmit temperature and depth data in pre-determined numerical bins: “YES” it was, “NO” it was not, “ND” tag not deployed
DATA_TS	for PAT tags, were the tags programmed to transmit temperate and depth data as a time series: “YES” it was, “NO” it was not, “ND” tag not deployed
TBL_cm	total body length in centimeters of the shark measured at original capture
SEX	sex of the shark determined by external morphology: “F” female, “M” male, “U” undetermined
INTERACTION	nature of the capture operation that obtained the shark for electronic tagging: “fishery, commercial” the shark was bycaught in a commercial fishery, “research” a dedicated scientific research operation
CAPTURE_GEAR	the fishery gear used to capture the shark by main category and subtype (for gillnets): “other” unspecified, “NC” not caught, jab tagged
TARGET FISHERY	for commercially bycaught sharks, the target species for the fishery operation: “NA” not applicable or available
LOCATION_REL	geographic place name of the nearest distance shore location from the at-sea release location
LAT_REL	latitude of the release location
LON_REL	longitude of the release location
DIST_KM	minimum simple linear surface travel distance for either PAT or SPOT tag deployment
LAT_END_PAT	latitude position for PAT pop up location (decimal degrees): “ND” tag not deployed, “DNT” tag did not transmit
LON_END_PAT	longitude position for PAT pop up location (decimal degrees): “ND” tag not deployed, “DNT” tag did not transmit
DIST_PAT	minimum linear distance between the release location and the end position in kilometers for the PAT tag: “ND” tag not deployed, “DNT” tag did not transmit
LAT_END_SPOT	latitude position for SPOT pop up location (decimal degrees): “ND” tag not deployed, “DNT” tag did not transmit
LON_END_SPOT	longitude position for SPOT pop up location (decimal degrees): “ND” tag not deployed, “DNT” tag did not transmit
DIST_SPOT	minimum linear distance between the release location and the end position in kilometers for the SPOT tag: “ND” tag not deployed, “DNT” tag did not transmit
COMMENTS	observer notes and comments about the deployment

This describes the fields in the supplemental file JWS_metadata.xlsx.

**Fig. 1:**
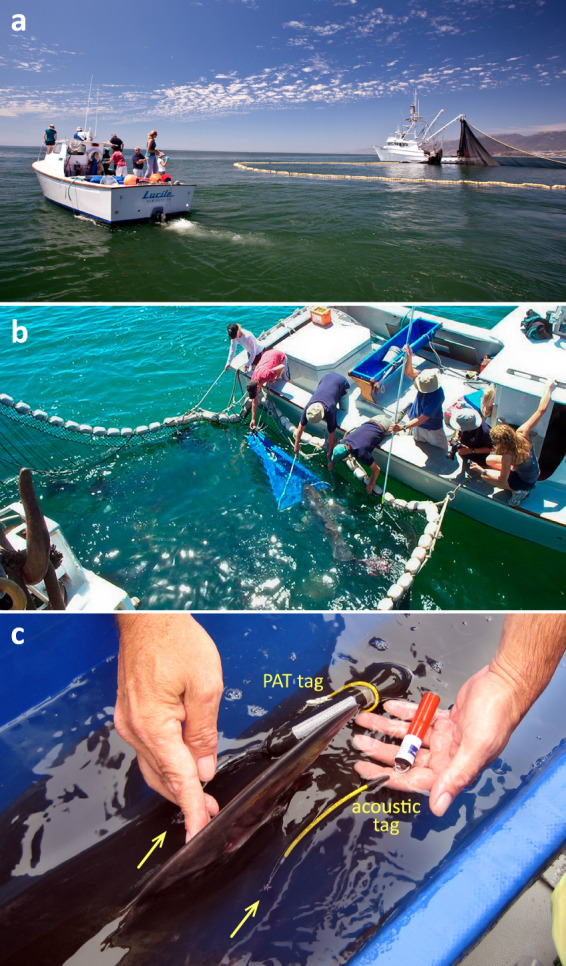
Depiction of a typical research operation for capturing and tagging juvenile White Sharks in the Southern California Bight. (**a**) Aquarium research vessel (RV Lucile) with crew approaching a contracted purse seine vessel containing a captured juvenile white shark. (**b**) Research crew on the RV Lucile leading the shark into a sling, where it is subsequently transferred to the vessel’s deck for tagging. (**c**) Successfully applied PAT and acoustic tags each positioned lateral of the dorsal fin, anchored via leaders, and affixed with titanium darts (yellow arrows). All images taken by Steve McNicholas (Great White Shark 3D) for the Monterey Bay Aquarium and used with permission.

**Fig. 2:**
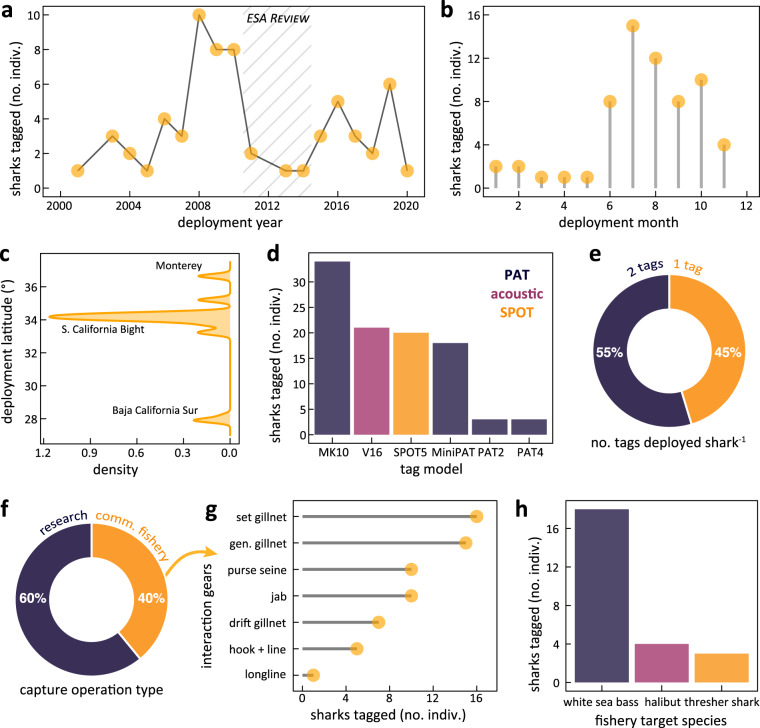
Metadata summaries of the field program that deployed biologging tags on juvenile white sharks in the southern California Current. (**a**) Deployment schedule for 72 electronic tags released on 64 White Sharks from 2001–2020 (**b**) Tagging activity peaked in the late summer months when the population is most locally abundant. Field operations decreased from 2011–2013 when the population was being considered for listing under the U.S. Endangered Species Act (ESA). (**c**) Deployments focused on opportunities in the Southern California Bight coastline and included deployments in the nursery area of Bahía Sebastian Vizcaíno, Mexico and releases after exhibition at the Monterey Bay Aquarium. (**d**) Researchers released a variety of pop-up archival transmitting (PAT, 58 sharks), acoustic (21 sharks), and smart position and temperature (SPOT, 20 sharks) tags. This manuscript only reports the geolocation, temperature and depth data from the PAT and SPOT platforms. (**e**) Half (35 of 64, 54.7%) of all sharks received multiple tags, primarily to compare their relative performance. (**f**) Most tags (38 of 64, 60.3%) were deployed during focused scientific research operations. (**g**) The remainder were joint operations resulting from opportunistic bycatch in commercial fisheries using various gears and (**h**) Targeting various species. “Jab” gear refers to research operations that uses pole extensions to apply tags to sharks without capturing and handling.

**Fig. 3:**
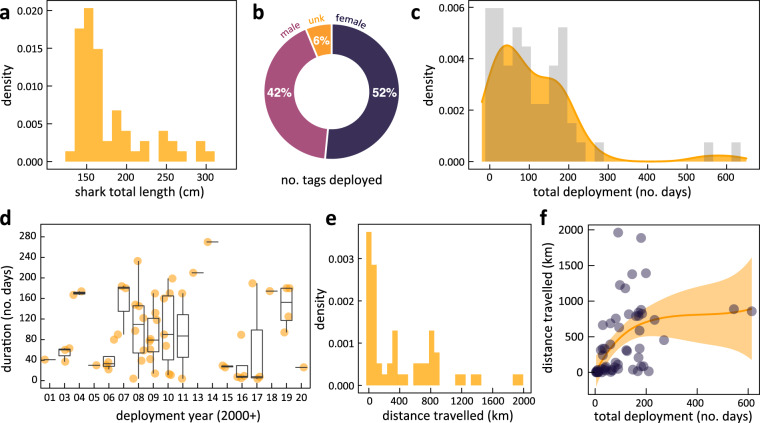
Demographic and deployment summaries from the juvenile white shark tagging program. (**a**) Total body length (TL) histogram indicates that most individuals tagged were either neonates (<1.50 m total body length “TBL”), young-of-year (YOY, <1.75 m TBL), or other juveniles (<2.5 m TBL) which typically inhabit the geographic region of focus^18,20,21,25^. This plot excludes one 396 cm TBL female that was opportunistically tagged off Santa Rosa Island. (**b**) Over half of the individuals (33 of 64, 51.6%) were females, with the sex undetermined for 4 individuals (6.3%) that were jab-tagged and not landed. (**c**) A majority of tags had a short deployment length which is the duration when the tag is logging data on the shark’s activity. (**d**) For 50 of 64 sharks (78.1%) this was less than 6 months, and less than 3 months for half of all sharks averaged for each deployment year represented as boxplots with the raw observations. (**e**) Histogram of straight linear distance between the release site and the location of first tag reporting. While many juvenile White Sharks did not travel far during tag deployments, there are notable exceptions. (**f**) Two juvenile sharks, for example, swam a linear distance of nearly 2,000 km, each in under 200 days. Solid orange line is a locally weighted regression (shaded area is standard error) which is influenced by location of release and annual migration cycles.

Figure 1 illustrates a typical *C. carcharias* tagging operation. This involves a contracted commercial fishing vessel with purpose-built gears to capture sharks (Fig. 1a) and a research crew to handle animals, monitor health (Fig. 1b) and attach electronic tags (Fig. 1c). More details on the tagging program and its methodologies are provided elsewhere^14,19,20^. Figure. 2 provides summaries of the deployment schedule, geographic locations, devices, and capture operations. Of note, 39.7% (25/64) of all tagging operations involved collaborations with commercial fishery operators (Fig. 2f–h), whose engagement was temporarily impacted (Fig. 2a) during the scientific review process when the population was under consideration for US Endangered Species Act listing. Figure 3 displays the demographic focus on small juvenile *C. carcharias*, with modest deployment durations and travel distances.

### SPOT5 platform sensors and configuration

Smart position and temperature transmitting (SPOT) tag data included in this dataset were obtained from SPOT5 tags (Wildlife computers, Redmond, WA) deployed on juvenile White Sharks between 2006 and 2009. These tags generated the locations of tagged sharks when the fin-mounted tag broke the surface of the water and there were Argos satellites overhead (http://www.argos-system.org). Several factors will influence the frequency of Argos locations from SPOT tags (hourly to weekly), as well as the accuracy of the positions (<250 m to >10 km), including: the sea state, the shark’s surface-oriented behavior, and the satellite coverage^23^. These factors determine how many messages from the tag reach the Argos satellite system, and therefore the location quality class. For instance, location class Z does not allow for a valid location to be estimated, classes B and A are assigned when only 2 and 3 messages are received, respectively and cannot estimate the accuracy of the location. Class 0, 1, 2 and 3 all require 4 messages to be received and have estimated errors of, respectively, over 1500 m (sometimes much greater), between 500 m and 1500 m, between 250 m and 500 m, and below 500 m. For additional technical details on location classes and position accuracy, see the CLS Argos User Manual (available at https://bit.ly/3uuMbzt).

The SPOT tags were programmed to only transmit location (and not time at temperature histograms or haul out statistics). They were also programmed to check if the wet/dry sensor is dry (and therefore the tag is out of the water and able to transmit) every 0.25 seconds. Messages sent by the tag were received by the Argos satellite system and transferred to the Wildlife Computers data portal from which data files were downloaded. The file formats included the proprietary .DIAG and .PRV file formats as well as a series of .CSV files. Location data are available within the locations.csv file for each tag.

### PAT platform sensors and configuration

Pop-up archival transmitting (PAT) tags deployed on juvenile White Sharks used to collect data for this dataset included MK10 (deployed 2003–2016), MiniPAT (deployed 2010–2020), PAT2 (deployed 2001–2003) or PAT4 (deployed 2004–2005) tags from Wildlife Computers (Redmond, WA). All PAT tag models included wet/dry, light level, pressure, and temperature sensors. These tags were programmed to collect light level, depth and temperature data while deployed on White Sharks, and at a pre-determined date, release from their anchor and float to the surface where they could transmit a subset of their data to the Argos satellite system.

Once at the surface, a final (pop-up) location of the tag is calculated by the Argos satellite system by measuring the “Doppler shift” of repeated transmissions received by the satellite as it moves over the tag. Multiple Argos locations are calculated in this way, and just as with SPOT tags described above, Argos quality classes are associated with each location. If tags reported on schedule, the first high quality location (class 1, 2 or 3) of the tag as determined by the Argos satellite is considered the final known location of the tagged shark.

While the battery remains sufficiently charged, the tag transmits packets of information to the Argos satellite system with the archived data from the light, pressure, and temperature sensors. Due to the combination of deployment length, battery life and satellite availability over the location of the tag, only a small percentage of the archived data will be able to be transmitted. For this reason, PAT tags can be programmed by the user to prioritize which data to transmit and in what format (e.g., full time series of depth or temperature vs. binned histograms of time spent at depth and temperature). Table 2 contains an overview of the fields in the metadata file (PAT_programming.xlsx) that reveals how the PAT tags used in this study were programmed. When a tag that has popped up could be recovered, the full archived data set was downloaded. This provided fine scale data on the depth, temperature and light level experienced by the tag, which were then uploaded to the Wildlife Computers data portal.

**Table 2:** Metadata descriptions of how the deployed tags were programmed.

FIELD	DESCRIPTION
SHARK_ID	unique identification number for each individual tagged shark
PAT_MODEL	Wildlife Computers model number for the PAT tag
PAT_ID	the unique serial number of the PAT tag
PAT_PTT	the PTT number for the PAT tag
PAT_DEPLOY_ID	user friendly unique PAT tag deployment ID, researcher assigned, matches ID listed within WC data files
PAT_MANUFACTURER_ID	manufacturer assigned unique PAT tag deployment ID, ATN internal reference ID: “DNT” indicates tag did not transmit
PAT_RECOVERY	was the PAT tag physically recovered? “YES” indicates it was, “NO” means it was not
PAT_DATA_TRANS	did the PAT tag transmit it’s data via satellite connection? “YES” indicates it did, “NO” means it did not
DATA_BINNED	for PAT tags, were the tags programmed to transmit temperature and depth data in pre-determined numerical bins: “YES” indicates it was, “NO” means it was not
DATA_TS	for PAT tags, were the tags programmed to transmit temperate and depth data as a time series: “YES” indicates it was, “NO” means it was not
SAMPLING_INTERVAL	how often the PAT tag sampled for depth and temperature (this may be different from the interval between observations in the time series file if the transmitted data were thinned): “NA” means not applicable or available
DATA_BINS_HRS	the duration (in hours) of each data bin for constructing the depth and temperature histograms: “NA” means not applicable or available
TEMP_BINS	the break points for the temperature bins for the histograms: “NA” means not applicable or available
DEPTH_BINS	the break points for the depth bins for the histograms: “NA” means not applicable or available
NOTES	notes about the programming details for each PAT tag

This describes the fields in the supplemental file PAT_programming.xlsx.

One of the most useful outputs of the PAT tag platforms are estimates of the location of the tagged shark while at liberty (between the known position at the time of release, and the known position at pop-up directly through the Argos satellite system). To estimate position while on the shark, the tag records light levels and produces two light-level curves each day of deployment. Using the onboard clock set to UTC time, the times of the local dawn and dusk are compared to UTC, which provides an estimate of the longitude of the tag on that day. The time between dawn and dusk (i.e., the length of the day) is used to estimate the latitude of the tag based on the day of the year. Both the latitude and longitude estimates are very much dependent on the quality of the light curves, which in turn are very dependent on the environmental conditions experienced by the tag (cloud cover, depth water turbidity, etc.). Higher quality light curves will produce more accurate geolocation estimates. This approach has been used for decades^19^ and has been independently validated^20^.

To further refine these geolocation estimates, the light-level data are processed through a proprietary geolocation algorithm on the Wildlife Computer portal called GPE3. The user provides an estimate of the average swimming speed of the tagged animal, and the GPE3 process employs a discretized Hidden Markov model that uses light levels, sea surface temperatures from satellites to compare with the onboard temperature recordings, and any known locations (such as the deployment and pop-up locations) to reduce the uncertainty around each daily geolocation estimate. More information about the GPE3 can be obtained from Wildlife Computers (www.wildlifecomputers.com).

### Data transmission and processing

Data from successful SPOT (*n* = 19) and PAT (*n* = 51) tag deployments were transmitted through Argos Services directly to the manufacturer and then decoded using their data analysis program (DAP; Wildlife Computers). Data from recovered archival tags (*n* = 26) were manually uploaded directly to the Wildlife Computers (WC) data portal by participating researchers and then decoded using DAP. Decoded raw telemetry data and when applicable processed GPE3 files (PAT tags only, see above) were then downloaded from the Wildlife Computers data portal to the ATN DAC via the Wildlife Computers API as .CSV files and in some cases in the proprietary WC file format using the unique manufacturer assigned deployment ids (Table 1). Downloaded data were zipped and maintained as is.

## Data Records

Researcher-assigned unique deploy identification numbers (i.e., Shark ID_PTT) were used to label each zip file (see Table 1). The subset of files included within each deployment folder are contingent on tag model, programming selections and whether a tag was successfully recovered. Individual data files, from successfully transmitted tags, regardless of tag type were prefixed by a tag’s assigned Platform Transmitter Terminal (PTT) id followed by the specific WC file type. Recovered archival PAT tag data files were prefaced with ‘out’ followed by the file type. Processed GPE3 files were prefixed with the deployments unique deploy identification number and the numerical suffix indicates researcher selected GPE3 file run. Unique deploy id, PTT id and tag type were provided within each individual data file to assist with future merging and reuse of these data. Blank cells contained within any of the provided data files signify attributes were not collected or determined. While data file and attribute descriptions are separately provided by the manufacturer, the full suite of animal and tag deployment metadata are fully described with an accompanying ISO 19115 metadata record for geospatial data.

Public access to the full data records and metadata from these 70 successfully transmitted and/or recovered electronic tags deployed on juvenile *C. carcharias* from 2001–2020 are available through the ATN data portal (https://bit.ly/2ZTvbFS) as well as the Research Workspace (RW) Data Observation Network for Earth (DataONE) member node (https://search.dataone.org/portals/RW). These data have a standard CC-BY license and a standalone, upstream Digital Objective Identifier (10.24431/rw1k6c3) specific to the dataset itself^23^. These deployment location files (i.e., location.csv or GPE3-X.csv) are also visualized within the ATN DAC data portal (project page, https://bit.ly/2YUlJ4P).

These data are publicly accessible and free to use without restriction, but we request future users of these data acknowledge the ATN as well as cite this data manuscript from which the data were obtained in any future publications and/or representations of these data.

## Technical Validation

### Post-processing of raw data

Raw data files were harvested directly from the tag manufacturer by the ATN DAC and preserved as is. Files were reviewed for completeness, and to ensure proper ids were provided and correct folder and file labels applied. However, it is strongly encouraged that users carefully review provided data files as well as read and fully comprehend associated metadata prior to use. The accuracy and precision of Argos derived location estimates are known to vary and device sensors can drift over time or even report erroneous results (outliers) due to data transmission errors. It is essential that prudent actions are taken to ensure data used in any future analyses are biologically sound and only include data from within each tags reported deployment window (see Table 1). This applies to both individual use of these data and aggregated use with other data.

## Usage Notes

Seven juvenile white sharks that were captured and tagged, were not immediately released, and their transmissions should be interpreted accordingly. Five of these sharks (6_10, 07_05, 08_11, 09_11B, 11_06) were displayed on exhibit at the Aquarium for a period of 22–198 days^24^. Three of these sharks were released back into the ocean in Monterey Bay (6_10, 07_05, 09_11B), which at the time, was significantly north of their natural habitat^18,20,25^. The remaining two exhibited sharks were released near Goleta, California at the northern edge of their historical range. Sharks 04_02 and 07_03 were kept in floating coastal pens (see Fig. 1a) for 6–8 days before being released. Shark 09_11 and 09_11B are the same individual, but only the tag from 09_11B reports data from the wild environment. The tag deployed on shark 09_11 while in the pen was not programmed to transmit and acted as a control. No exhibited sharks carried tags while on display. Shark 09_09 and 09_09B are also the same individual. Shark was 09_09 was recaptured and tagged with a new PAT tag at which point it was also given an updated shark id (09_09B). Additional context on the future application of these data to understand animal movements, migrations, habitat preferences, niche modeling, mortality, and climate change impacts are provided in studies published with partial subsets of the present data^13–22^.

## Supplementary information

JWS_metadata.xlsx

PAT_programming.xlsx

## Data Availability

All the data and code used in this study are available open access from the ATN DAC Data Portal (https://portal.atn.ioos.us) and the Research Workspace DataONE member node (https://search.dataone.org/portals/RW) as well as at GitHub (https://bit.ly/3noJCJD). As we are providing the raw telemetry data and metadata from the platform manufacturer, the code we provide is for data visualization used to make the figures in this manuscript.
